# Effect of Various Irrigating Devices on the Removal of Two Different Forms of Calcium Hydroxide from Internal Resorption Cavities

**DOI:** 10.1155/2020/8881177

**Published:** 2020-09-12

**Authors:** Nima Mousavi Nasab Mobarakeh, Afsaneh Taheri, Hadi Rahmanian, Dana Jafarpour, Sareh Rahmanian

**Affiliations:** ^1^Department of Endodontics, School of Dentistry, Shiraz Azad University, Shiraz, Iran; ^2^School of Dentistry, Shiraz Azad University, Shiraz, Iran; ^3^University of Catholique, Lyon, Rhone-Alpes, France; ^4^Biomaterials Research Center, School of Dentistry, Shiraz University of Medical Sciences, Shiraz, Iran

## Abstract

The present study aimed to investigate the efficiency of passive ultrasonic irrigation (PUI), EndoActivator (EA), standard needle irrigation (SNI), and XP-endo Finisher files in removing pure calcium hydroxide (pCH) and injectable CH from *in vitro* root resorption cavities. Using a rotary system, the root canals of 116 extracted single rooted teeth were prepared. Imitated internal resorption cavities were then created in root halves. The specimens were divided into two groups according to the form of CH (*n* = 58): (I) pCH; (II) injectable CH. The teeth of each group were randomly divided into six subgroups: negative control (*n* = 5), positive control (*n* = 5), PUI (*n* = 12), XP-endo Finisher (*n* = 12), EA (*n* = 12), and SNI (*n* = 12). The root canals were irrigated using NaOCl and EDTA and split longitudinally, and both halves were evaluated with a stereomicroscope. Kruskal–Wallis and Mann–Whitney *U* tests were used to analyze data. The present results revealed that PUI completely removed pCH in 79% and injectable CH in 70.8% of the internal resorption cavities which was significantly higher than other methods (*p* < 0.05). There was no statically significant difference between different forms of CH in terms of CH removal (*p*=0.918). The PUI technique was observed as the most efficient method of P-CH and injectable CH removal from a replicated internal resorption cavity. Finally, according to the findings, different forms of CH were comparable in terms of CH removal.

## 1. Introduction

Successful removal or reduction of microorganisms and their products from root canals is the determining factor in prognosis of teeth with pulp necrosis and apical periodontitis [1, 2]. In long-lasting infection, bacteria might colonize the entire root canal system including ramifications, isthmuses, and dentinal tubules from which they are scarcely removed by chemomechanical interventions [3, 4].

There have been significant improvements in instrumentation procedures throughout the years; however, none of the present methods are capable of disinfecting the root canal structure [5]. Hence, the placement of an intracanal medicament with extensive antibacterial activity is required to recover root canal disinfection and to optimize treatment outcome [6–8]. In this respect, calcium hydroxide (CH) is among the most common intracanal dressings which has been widely accepted in endodontic therapy used in multisession endodontic treatments to disinfect the canal and terminate the resorption process. Traditionally, calcium hydroxide paste was obtained by mixing pure powder with distilled water (DW) or saline and was transferred into the canal by means of various techniques, remaining in the root canal system for several days or weeks [7, 9].

To avoid negative interactions between the filling material and the intracanal dressing and to maximize sealer adaption to the root walls, the temporary medicament should be removed completely prior to root canal obturation [10]. Conventionally, intracanal calcium hydroxide was removed by instrumenting using a master apical file and irrigating abundantly using sodium hypochlorite or ethylenediaminetetraacetic acid (EDTA) [11, 12].

On the other hand, canal irregularities, which are inaccessible for conventional irrigation procedures, make complete removal of CH difficult and residues often remain on the root canal surface particularly in the apical part [11–13]. It has been stated that residual CH influences dentine bond strength [14, 15], affects the infiltration of sealers to dentinal tubules [16] which might cause apical microleakage, and, thereby, compromises treatment success [11, 17, 18]. Therefore, several methods have been introduced to maximize the efficiency of CH paste removal from root canal system [19].

Passive ultrasonic irrigation (PUI) is an irrigation technique in which the irrigant is passively agitated inside the canal via an ultrasonic tip which is placed inside the canal and is passively moved up and down to the working length (WL). This technique was designed with an aim to enhance the efficiency of canal decontamination [20]. Previous studies have reported that PUI is more efficient in removing debris from the canal surface compared to the conventional irrigant syringe delivery [21, 22].

The EndoActivator (EA) is another system which has been introduced for an attempt to increase the irrigation effectiveness. EndoActivator is a cordless handpiece and a sonic motor. By means of this system, safe intracanal solution activation might be accomplished through dynamic intracanal fluid agitation [23].

Recently, a new nickel-titanium rotary finishing file named the XP-endo Finisher file has been presented. According to the manufacturer's claims, the XP-endo Finisher file is suggested for application following root canal instrumentation in order to achieve an optimum root canal disinfection and at the same time preserve dentin structure [24, 25].

While there are studies in literature on the effectiveness of different irrigation methods in removing CH from the root canals, no study has compared the efficacy of these CH removal techniques in removing different forms of CH. Accordingly, the main objective of the present *in-vitro* investigation was to compare the efficacy of four removal methods of calcium hydroxide paste (passive ultrasonic irrigation, standard needle irrigation, EndoActivator, and XP-endo Finisher file) in removing different forms of CH (pure calcium hydroxide (pCH) and injectable CH) from the replicated internal root resorption. The null hypothesis was that there would be no difference between CH removal techniques in terms of removing neither pCH nor injectable CH from simulated resorption cavities.

## 2. Materials and Methods

The present research was approved by the local ethics committee (#16310201952014). A total of 116 human single rooted maxillary central incisors which were extracted due to periodontal reasons were chosen in this study. Written informed consent was obtained from the parents or guardians at the time of tooth extraction. The parents were informed about the purpose of the study, privacy preservation, and data anonymity. Teeth with any sign of resorption, decay, immature apex, previous restoration, crack, or fracture were excluded. To ensure the existence of a single root canal devoid of prior root canal treatment, calcifications, or resorption, radiographs of mesiodistal and buccolingual directions were taken. After extraction, any remaining soft tissues were removed from the tooth surface and the specimens were kept in a 0.1% thymol suspension (pH = 7) at 4°C for 30 d. To prevent dryness, the teeth were kept at ambient temperature with 100% humidity. In order to standardize the specimens, 20 mm from the apex was marked by a digital calliper (Teknikel, Istanbul, Turkey) and then the remaining crown was cut using diamond disks (Jota Co., Ruthi, Switzerland) under water cooling to obtain the desired root length for each tooth. The specimens were again kept at 100% humidity until the experimental procedures. Then, a size 15 K-file (Mani Inc., Tochigi-Ken, Japan) was positioned in the canal only to be detectible at the apical foramen. By subtracting 1 mm from this measured length, the WL was recorded.

The ProTaper rotary system (Dentsply Tulsa, Tulsa, OK) was used up to size F5 (size 50, 0.05 taper) as the master apical file to instrument the 232 root canals. Three mL of 5.25% sodium hypochlorite (NaOCl) solution was used to irrigate the canal by means a syringe and a 30-gauge needle (Endo irrigation needle side vented, endo-top, Cerkamed, Poland) between each filing. Five mL of 17% ethylenediaminetetraacetic acid (MASTER-DENT EDTA solution 17%, Carolina, USA) was applied for 1 min to irrigate the root canals after final preparation. Finally, 10 mL of distilled water was applied for irrigation and the root canals were dried using paper points (Absorbent Paper Points; Meta Biomed Co. Ltd., Baotou, China). Afterwards, the teeth were embedded in modified Eppendorf vials using silicone impression material (Coltene Whaledent, Altstätten, Switzerland). The specimens were then removed from the impression material. Longitudinal grooves on the buccal and lingual surfaces were made using a diamond bur (Jota Co., Ruthi, Switzerland) under cooling water. The specimens were then fragmented in a buccolingual direction along their long axis by means of a cutting machine. Using a digital calliper, the root extension was recorded. The root length was measured and the middle-third area was encircled in each segment to make sure that a same-level resorption was replicated in both halves. In order to standardize the artificial resorption cavities, 5 mm from the apex was marked using a digital calliper (Teknikel, Istanbul, Turkey). Then, the artificial internal resorption cavities with 0.8 mm in depth and 1.6 mm diameter were prepared 5 mm above the apex using a round bur (Jota Co., Ruthi, Switzerland). Finally, the two halves were placed next to each other in the primary position. To attain a close fitness of the root halves, Super Glue (Sanabond glue, Geg 1, 2, 3, Iran) was applied and the teeth were remounted in Eppendorf vials. To ensure an open root canal pathway, a size 40 K file was used.

The specimens were divided into two groups based on the form of CH: (I) calcium hydroxide mixed with distilled water (pCH) (*n* = 58); (II) injectable paste: a commercial preparation, preloaded in carpules (Multi-Cal calcium hydroxide paste, PULPDENT, USA) (*n* = 58).

In each group, five teeth were left untreated as the negative control group. Nothing was applied to the canals of the negative control groups. For the rest of the specimens in group I, CH powder (Calcium hydroxide powder, Golchadent, Iran) was spatulated with DW at a powder to liquid ratio of 1 : 1.5. Size 40 of lentulo spiral carriers (in a low-speed handpiece with a moderate speed) were used to insert the CH paste in a way that the paste overtruded through the foramen. In group II, the water-soluble Multi-Cal CH was directly applied by means of a syringe needle 1 mm short to the WL; then, backfilling was performed. A radiograph was taken following CH placement to make sure that the entire canal was consistently filled with CH up to the WL.

A temporary filling material (Cavit, ARIA Dent, Coltosol, Iran) was used to seal the endodontic access cavities. The specimens were then kept at 37°C at 100% humidity for 1 week. After removing the temporary fillings, a size 15 K file (Dentsply Maillefer) was implemented to the working length for CH loosening and creating space for the irrigation needle.

In each group, five teeth were appointed as the positive control group. These teeth were filled with CH without any removal procedure. Then the remaining 48 teeth in each group were randomly divided to four subgroups based on the CH removal technique (*n* = 12): PUI, XP-endo Finisher, EA, and SNI.

### 2.1. Experimental Groups and CH Removal Protocols

#### 2.1.1. Passive Ultrasonic Irrigation Group

Five mL of 17% EDTA and 5 mL of 5.25% NaOCl were passively agitated in this group by means of an ultrasonic tip (EMS, Le Sentier, Switzerland). An ultrasonic file (size 20, 0.02 taper) (U file, Varios, NSK, Japan) was located in the canal to 1 mm shorter than the WL, avoiding to touch the walls and activated at 1 min for either solution. Each ultrasonic file was used for three root canals. Finally, each specimen was washed with 5 ml of saline.

#### 2.1.2. XP-Endo Finisher Group

In this group, 5 mL of 17% EDTA and 5 mL of 5.25% NaOCl were each applied via a syringe and a 30-gauge needle (Navi-Tip; Ultradent, South Jordan, UT, USA). The WL was adjusted using a rubber-stop. The XP-endo Finisher (XP-end Finisher, FKG swiss endo, Switzerland) was chilled using the cooling spray and rotated out of the tube with a lateral movement. The rotation was stopped after the file was removed. This procedure was performed to ensure that the file was straight. The XP-endo Finisher file was applied to the canals filled with irrigant, while it was straight. In accordance with the manufacturer's instructions, the file was set at 900 rpm and 1 N·cm torque and was advanced to the WL. Slow and gentle 7-8 mm lengthwise movements were made for 1 min. The XP-endo Finisher was activated at 1 min for each solution. Each XP-endo Finisher was used for one root canal. Finally, each specimen was washed with 5 ml of saline to remove debris and residual material.

#### 2.1.3. EndoActivator (EA) Group

Each canal was filled with 5 mL of 5.25% NaOCl and then EA (Dentsply maillefer, CH-1338 Ballaigues, Switzerland) with a medium tip size of 25 and 0.04 taper was inserted into the canal to 1 mm short of the WL and activated for 1 min at 10 000 cpm. Five ml of 17% EDTA was then introduced into the canals, and EA was again activated for 1 min. Each EA tip was used to prepare one root canal. Finally, each tooth was washed with 5 ml of saline to remove debris and residual material.

#### 2.1.4. Standard Needle Irrigation (SNI) Group

For the removal of CH from simulated internal resorption cavities, irrigation was performed with 5 mL of 5.25% NaOCl for one min, followed by 5 mL of 17% EDTA for one min, using a syringe and a 30-gauge needle (Navi-Tip; Ultradent, South Jordan, UT, USA) with 2 to 6 mm vertical motions placed 1 mm short of the WL without touching the walls. No additional agitation of irrigants was performed. Finally, each specimen was washed with 5 ml of saline.

### 2.2. Image Evaluation

**Figure 1:**
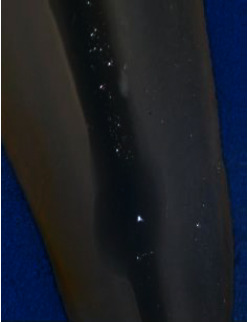
Score 0. The cavity is empty (e.g., in the negative control group).

**Figure 2:**
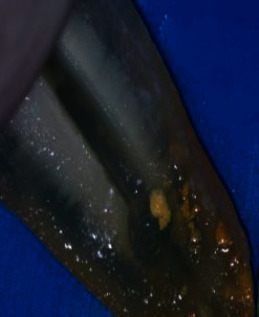
Score 1. Less than half of the cavity is filled with CH remnants.

**Figure 3:**
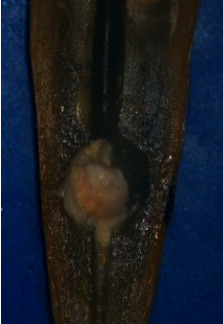
Score 2. More than half of the cavity is filled with CH remnants.

**Figure 4:**
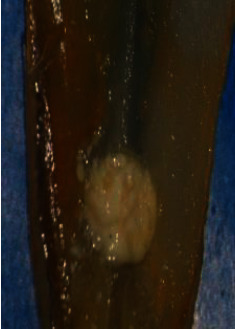
Score 3. The complete cavity is filled with CH remnants (e.g., in the positive control group).

Following irrigation, the root halves were split. Digital images were captured of the replicated internal resorption cavities at 25 × magnification using a digital camera (BestScope, BS-3060C, China) attached to a stereomicroscope (BestScope, BS-3060C, China). Two standardized examiners scored the CH remained in the replicated resorption cavities in both halves following irrigation using the following score system [26] (Figures 1–4):

- 0 = the cavity is empty (Figure 1)
- 1 = less than half of the cavity is occupied with CH remnants (Figure 2)
- 2 = more than half of the cavity is occupied with CH remnants (Figure 3)
- 3 = the complete cavity is occupied with CH remnants (Figure 4)

### 2.3. Statistical Analysis

Data analysis was conducted using SPSS 20 software (SPSS Inc., Chicago, IL, USA). The Kolmogorov–Smirnov test was used to measure normality and revealed a nonnormal data distribution. The variances in CH scores between the subgroups within each group and the CH scores between two forms of CH were analysed by Kruskal–Wallis and Mann–Whitney *U* tests, respectively. Intraexaminer agreement was calculated by using Cohen's Kappa coefficient. The significance level was set at *p* < 0.05.

## 3. Results

The results of CH removal scores of different removal techniques in group I are shown in Table 1.

In group I (pCH), there was a significant difference between various removal methods in terms of CH removal (*p* < 0.001). In this group, the PUI technique achieved complete clearance of pure calcium hydroxide paste in 70.8% of cases (0 score) which was significantly higher than other removal techniques (*p* < 0.05). There was no significant difference between negative control and PUI in terms of complete CH removal (*p*=0.325).

After PUI, the highest efficiency in pure calcium hydroxide removal was in XP-endo Finisher (45.8%) which was significantly higher than EA and SNI groups (*p*=0.002 and *p* < 0.001, respectively). No significant difference was observed between EA and SNI groups in terms of complete CH removal (*p*=0.630).

The results of CH removal scores of different removal techniques in group II are revealed in Table 2.

In group II (Injectable CH), there was a significant difference between various removal methods in terms of CH removal (*p* < 0.001). In this group, the complete clearance of pure calcium hydroxide paste (score 0) was observed in 70.8% of cases of PUI technique which was significantly higher than other removal techniques (*p* < 0.05). There was no significant difference between negative control and PUI in terms of complete CH removal (*p*=0.432).

The highest efficiency in injectable calcium hydroxide removal after PUI was in XP-endo Finisher (45.8%) which was significantly higher than SNI groups (*p* < 0.001). No significant difference was observed between XP-endo Finisher and EA groups in terms of complete CH removal (*p*=0.732).

In addition, the results of Mann–Whitney *U* test revealed that there was no significant difference between different forms of CH (groups I and II) in terms of CH removal (*p*=918). The type of CH made no significant difference in positive control (*p*=1.000), negative control (*p*=1.000), PUI (*p*=0.530), EA (*p*=0.075), SNI (*p*=0.556), and XP-endo Finisher (*p*=0.396) groups.

## 4. Discussion

Nowadays, for ease of use and to increase the speed, premixed injectable calcium hydroxide syringes have been introduced. Compared to the pure CH, the premixed CH contains substances such as methyl cellulose matrix and radio-opacifying materials such as barium sulfate. Both types of calcium hydroxide have high pH and possess antimicrobial properties, but so far no study has been performed on the difference in the removal of these two types of calcium hydroxide from the root canal.

Dental practitioners routinely remove calcium hydroxide from the root canal using instrumentation with the master apical file in combination with abundant irrigation [11, 12] which have been proven ineffective by numerous studies [11–13, 26]. It has been reported that traditional needle irrigation cannot completely purify many inaccessible areas such as isthmus, internal resorption areas, and especially the apical third of the canal. Thus, canal irregularities may be inaccessible for conventional irrigation procedures and CH may remain in these extensions [26–28].

Calcium hydroxide retention in these areas can block dentinal tubules and interfere with sealer adhesion to dentin which may eventually lead to increased microbial leakage and, thereby, result in long-term failure [17, 18]. In a study by Balvedi et al. [28], it was observed that after using different irrigation techniques, the percentage of residual calcium hydroxide in the cervical and middle thirds was significantly lower than that of the apical thirds. They also verified better results with PUI technique. According to Balvedi et al., this finding might be related to the anatomical differences in the apical third and the accessibility of the devices to the apical's narrow space [28]. Accordingly, the location of the simulated internal resorption holes in the present study was generated in the apical third.

Nowadays, new techniques have been proposed to increase the efficiency of canal irrigations including passive ultrasonic irrigation (PUI), ultrasonic irrigation, and the new XP-endo Finisher file. The manufacturers claim that these new systems are able to access and clean the remote and intact sites of canal. In our study, the efficacy of PUI, XP-endo Finisher, SNI, and EA methods was analysed in the removal of two types of pure (powder-liquid) and premixed injectable calcium hydroxide from resorption simulated cavities. The results of the current study showed that none of the proposed techniques were able to completely remove calcium hydroxide from all specimens. Consistent with previous findings [29–33], the results were similar for both pure and injectable calcium hydroxide groups; Taşdemir et al. [34] previously reported that none of their tested methods including XP-endo Finisher, canal brush, PUI, Endoactivator, and syringe needle irrigation were able to completely remove calcium hydroxide from internal resorption cavities. In line with our findings, the authors also showed that the XP-endo Finisher and PUI were more effective than irrigation alone. Wigler et al. [35] also verified that none of the studied methods could completely remove calcium hydroxide from standard synthetic fissures in the apical third of canal. They also reported that there was no significant difference between the XP-endo Finisher and PUI and both removed greater amount of calcium hydroxide than SNI. Accordingly, due to the anatomical complexity of the roots, novel canal cleansing methods and further studies on this topic are required.

Based on the results of this study, PUI was able to completely remove calcium hydroxide from internal resorption cavities in 79.2% of the injection group and 70.8% of pure powdered calcium hydroxide group which was significantly higher than other methods. In pure calcium hydroxide group, the highest performance after PUI was attributed to XP-endo Finisher (45.8%), EA (16.7%), and SNI (16.7%), respectively (*p* < 0.05). In the injectable group, after PUI, the highest performance was related to XP-endo Finisher (45.8%), EA (20%), and SNI (8.3%), respectively. Therefore, in line with the previous studies [29, 33, 36, 37], it was concluded that PUI possesses a great aptitude in clearing remote areas.

In a study by Jiang et al. [38], it was suggested that the higher velocity and volume of irrigant induced by PUI might explain its capability in calcium hydroxide removal from the root canal. Moreover, much evidence has shown that the superiority of passive ultrasonic over sonic energy, the cavitation effect, and eddy currents produced during the use of PUIs, as well as the bubbles created in the irrigants due to the transmission of energy by ultrasonic waves, are among the factors which led to the dominance of PUI technique [38].

In addition, the efficiency of XP-endo Finisher was evaluated in this study. It has been claimed that the XP-endo Finisher can remove hard tissue debris and smear layer from root canal system. Because of its favorable flexibility, ability to adapt to the root canal, and shape memory effect, the manufacturer recommends that calcium hydroxide clearance can be effectively achieved.

According to the manufacturer, the XP-endo Finisher, which is formed using a proprietary NiTi alloy (Martensite-Austenite Electropolish-FleX), has a small core size (ISO 25 in diameter and zero taper) with improved flexibility and performs at different temperatures. It has also been reported that XP-endo Finisher curved bulb can expand its extent 6 mm in diameter when the file tip is squeezed or 100 times of a corresponding sized file [24, 25]. The manufacturers claim that this new Finisher file is able to access and clean the remote and intact sites of canal.

The present results showed that the new XP-endo Finisher file has an acceptable potency to purify remote areas and is superior to SNI and EA in this respect. This finding is in agreement with that of Elnaghy et al. [32] and Keskin et al. [33] who also observed the XP-endo Finisher method to be more effective than the other tested methods. Nevertheless, in the injectable premixed calcium hydroxide, there was no significant difference between the XP-endo Finisher and the EA. Due to the novelty of this instrument, few studies have been conducted on its efficiency and, therefore, further research on the quality and performance of this file is needed.

There was no significant difference between the calcium hydroxide removal methods in the study conducted by Elnaghy et al. [32]. Of the tested CH removal methods including CanalBrush (CB), EndoActivator, PUI, and SNI, the authors suggested that the XP-endo Finisher and PUI methods might be more effective in eliminating the smear layer than the EndoActivator, CB, and SNI methods. Moreover, Elnaghy et al. [32] observed no significant difference between XP-endo Finisher and EA which was consistent with our findings for the pure calcium hydroxide group; however, this result was not observed with the premixed injectable group.

In an investigation by Keskin et al. [33], PUI and XP-endo Finisher were superior to SI. However, there was no significant difference between PUI and the XP-endo Finisher, which was not consistent with the findings of the current study.

Previously, a few studies evaluated the use of EndoActivator in the removal of calcium hydroxide from the canal walls. Our findings revealed no significant difference between the EA and SNI groups in the powdered calcium hydroxide group and between the EA and XP-endo Finisher groups in the premixed calcium hydroxide. Keskin et al. [33] also found no significant difference between EA and SNI, which is consistent with the results of the present study in the pCH group. One of the causes of EA inferiority compared to the PUI technique is the inability of this method to produce cavitation and the lower potency of sonic compared to ultrasonic [33].

According to the results of the current study, all modern removal methods are significantly more effective than the traditional instrumentation with the master apical file in combination with copious irrigation method and, thus, are recommended to increase the prognosis of treatment. In this study, the removal efficiency of each method was also compared between the pure and injectable calcium hydroxide groups, indicating that there was no significant difference in CH removal between different forms of CH. This result suggests that in spite of changes in viscosity and concentration of calcium hydroxide, the addition of substances such as methyl cellulose and barium substrate had no effect on CH removal by the studied methods. However, given the differences in the proportion and content of syringes introduced by different manufacturers, further studies are recommended to substantiate data observed in this research.

## 5. Conclusion

None of the tested techniques were able to completely remove CH from resorption cavities. PUI was the most effective method for removal of P-CH and injectable CH from a simulated internal resorption cavity. Different forms of CH were comparable in terms of CH removal.

## Figures and Tables

**Table 1:** Comparison of removal efficacy between CH removal methods in group I.

Groups	Number (%)				Total
	Score 0	Score 1	Score 2	Score 3	
Negative control	10 (100)^A^	0 (0)	0 (0)	0 (0)	10 (100)
Positive control	0 (0)^D^	0 (0)	0 (0)	10 (100)	10 (100)
PUI	17 (70.8)^A^	6 (25)	1 (4.2)	0 (0)	24 (100)
XP-endo Finisher	11 (45.8)^B^	8 (33.3)	2 (8.3)	3 (12.5)	24 (100)
EA	4 (16.7)^C^	5 (20.8)	9 (37.5)	6 (25)	24 (100)
SNI	4 (16.7)^C^	6 (25)	4 (16.7)	10 (41.7)	24 (100)

Different uppercase letters show significant difference between groups (in a column).

**Table 2:** Comparison of removal efficacy between CH removal methods in group II.

Groups	Number (%)				Total
	Score 0	Score 1	Score 2	Score 3	
Negative control	10 (100)^A^	0 (0)	0 (0)	0 (0)	10 (100)
Positive control	0 (0)^D^	0 (0)	0 (0)	10 (100)	10 (100)
PUI	19 (79.2)^A^	4 (16.7)	1 (4.2)	0 (0)	24 (100)
XP-endo Finisher	11 (45.8)^B^	8 (33.3)	2 (8.3)	3 (12.5)	24 (100)
EA	5 (20.8)^B^	12 (50)	4 (16.7)	3 (12.5)	24 (100)
SNI	2 (8.3)^C^	6 (25)	5 (20.8)	11 (45.8)	24 (100)

Different uppercase letters show significant difference between groups (in a column).

## Data Availability

The data used to support the findings of this study are included within the article.
